# Implicit bias to food and body cues in eating disorders: a systematic review

**DOI:** 10.1007/s40519-020-00974-9

**Published:** 2020-08-08

**Authors:** Georgios Paslakis, Anne Deborah Scholz-Hehn, Laura Marie Sommer, Simone Kühn

**Affiliations:** 1 Toronto General Hospital, University Health Network, Toronto, ON M5G 2C4 Canada; 2 Department of Psychiatry, University of Toronto, Toronto, ON M5T 1R8 Canada; 3 Department of Psychosomatic Medicine and Psychotherapy, Christian-Albrechts-University, Niemannsweg 147, 24105 Kiel, Germany; 4 University Medical Center, Hamburg-Eppendorf, Clinic and Policlinic for Psychiatry and Psychotherapy, Martinistraße 52, 20246 Hamburg, Germany; 5 Department of Psychosomatic Medicine and Psychotherapy, University Hospital of Erlangen, Schwabachanlage 6, 91054 Erlangen, Germany

**Keywords:** Anorexia nervosa, Bulimia nervosa, Binge eating disorder, Approach avoidance task, Implicit, Bias

## Abstract

**Background:**

Rigid, restrictive eating patterns, fear of gaining weight, body image concerns, but also binge eating episodes with loss of control leading to overweight, at times followed by compensatory measures to control weight, are typical symptoms in eating disorders (EDs). The regulation of food intake in EDs may underlie explicit processes that require cognitive insight and conscious control or be steered by implicit mechanisms that are mostly automatic, rapid, and associated with affective—rather than cognitive—processing. While introspection is not capable of assessing implicit responses, so-called indirect experimental tasks can assess implicit responses underlying a specific behavior by-passing the participant’s consciousness. Here, we aimed to present the current evidence regarding studies on implicit biases to food and body cues in patients with EDs.

**Methods:**

We performed a systematic review (PRISMA guidelines). We included controlled studies performed in clinical ED cohorts (vs. healthy control subjects or another control condition, e.g., restrictive vs. binge/purge AN) and using at least one indirect assessment method of interest.

**Results:**

Out of 115 screened publications, we identified 29 studies fulfilling the eligibility criteria, and present a synthesis of the essential findings and future directions.

**Conclusion:**

In this emerging field of research, the present work provides cornerstones of evidence highlighting aspects of implicit regulation in eating disorders. Applying both direct (e.g., self-reports) and indirect measures for the assessment of both explicit and implicit responses is necessary for a comprehensive investigation of the interplay between these different regulatory mechanisms and eating behavior. Targeted training of implicit reactions is already in use and represents a useful future tool as an add-on to standard psychotherapeutic treatments in the battle against eating disorders.

**Evidence level:**

1 (systematic review).

## Introduction

The terms implicit vs. explicit determine the degree of automatism of a particular response to specific cues [1]. An explicit response to a cue is target oriented and, thus, subject to attentional or strategic control and conscious awareness. All responses that are rapid/automatic, spontaneously induced and most probably lie outside a person’s awareness are implicit. The interplay between implicit and explicit responses forms behavior, e.g., food intake. In the realm of food intake, physiological (homeostatic) mechanisms are implicit [2]. Deliberate attempts to psychologically override natural regulatory processes like in restricted food intake (“diet”) are by definition explicit.

Direct methods, mostly self-reports upon request, assess explicit responses. While introspection is not capable of assessing implicit responses, so-called indirect experimental tasks can assess implicit responses underlying a specific behavior without the need to access the participant’s consciousness [1]. In such tasks, typically response times (i.e., the time between presentation of a cue and the actual response) serve as a surrogate for the strength of the association between cue and response. Thus, faster response times are indicative of a stronger cue-to-response association. Differences in response times, e.g., when it comes to the experimental approach vs. avoidance of cues, are defined as implicit (approach or avoidance) biases.

Direct, explicit measures are a necessary tool in behavioral and healthcare research as they enable quick and easy data collection in large samples and may also be used to measure constructs otherwise difficult to obtain by means of behavioral or physiological measures (e.g., introversion as a trait). On the other hand, response bias in explicit data collection is a well-known and widely discussed phenomenon. Beside the multiple reasons for which self-reported attitudes and behaviors may be biased (reduced introspective ability, cognitive distortions due to the clinical condition, e.g., underweight patients with Anorexia Nervosa feeling “fat” or denying having fat phobia, social desirability bias, or recall bias), response bias is subject to change following an intervention. This “response-shift bias” [3] occurs when an intervention changes an individual’s understanding or awareness of the target concept [4], thus affecting the bias at each measurement point. Also, behaviors may be influenced by the process of self-monitoring per se and could operate differently among those who are not self-monitoring their behaviors. In this sense, studies have reported that self-monitoring of exercise behaviors and physical activity levels are positively associated [5]. Based on dual-process theory [6], implicit and explicit bias capture different underlying processes, which drive different behavioral manifestations [7, 8]. Thus, even though explicit tools such as self-report questionnaires or the ecological momentary assessment (EMA) may help collect significant amounts of data, when possible, examining both types of biases may shed light onto how normative behavior (eating behavior) may become non-normative (e.g., restrictive eating). Discrepancies between implicit and explicit biases have been associated with unhealthy eating behavior and disinhibited eating [9, 10]. Implicit and explicit biases may independently predict behavior [11]. Several studies have examined implicit biases to food in different phenotypes among healthy cohorts (dieters vs. non-dieters, high food cravers vs. low food cravers, etc.), and found discrepancies between explicit and implicit biases. Restrained eaters displayed a stronger positive implicit bias to high-calorie foods as opposed to their negative explicit evaluations [12, 13]. A study comparing restrained to unrestrained eaters found a stronger approach bias to high-calorie food in restrained eaters [14]. Restricting food intake for the sake of a diet, a paradigmatic explicit behavior of cognitive control, goes along with enhanced cortical activity [15]. On the other hand, breaking a diet (“diet failure”) has been associated with an increase in the (implicit) reward sensitivity of food [16]. Such findings could be used to explain disinhibited eating in dieters who occasionally break their diet. Regarding emotional eating, indirect assessment methods have also shown a differential implicit bias pattern to food cues between high and low emotional eater [17, 18].

Next to indirect assessment studies using food cues, there are also studies that applied body-related cues and demonstrated implicit pro-thin/anti-fat bias [19–21]. These biases were associated with higher levels of disordered eating [20–22]. In fact, implicit biases were predictive of eating disorder (ED)-related symptomatology above and beyond the corresponding explicit biases [22]. Finally, in females with self-reported ED-traits, symptoms regarding body dissatisfaction were consistently associated with both explicit [23] and implicit [24] negative biases to food.

Thus, several studies showed differential patterns of implicit vs. explicit biases to food and body cues in non-clinical cohorts and associated these patterns with ED-related psychopathology. It is, therefore, not surprising that implicit and explicit aspects of eating behaviors have steadily found their way into clinical contexts as well. It was hypothesized that implicit biases and discrepancies to explicit ratings might be able to explain some of the key clinical characteristics of patients with EDs. Patients with Anorexia Nervosa (AN) display a high degree of self-control that helps them keep an explicit dieting goal even in the presence of a strong physiological urge to approach food [25]. Some authors have suggested that food has lost its incentive (implicit) value in patients with the restrictive type AN [26]. The regulation of the drive to intake food is also impaired in obesity, however in the other direction, with the rewarding properties of food being unusually high [27]. Patients diagnosed with Bulimia Nervosa (BN) or Binge Eating Disorder (BED) show frequent disruptions in inhibitory control leading to binge eating episodes despite their efforts to withdraw from such behaviors. In the case of BN and BED in particular, self-regulation abilities are hampered in situations of high emotional intensity (e.g., confrontation with stressors) or when the experienced stimuli are particularly strong (e.g., due to the availability of high palatable and rapidly available food) and disable counter-regulatory, implicit or explicit, mechanisms [28].

In this review, we systematically assessed the current evidence on implicit biases in EDs regarding food and body cues. Although there is an increasing number of studies examining implicit biases in ED cohorts, these studies are mostly cross-sectional and methodologically heterogeneous. In contrast to a recently published review that focused only on visual attentional biases in individuals with EDs [29], our work pursued a broader approach to this field by considering all available indirect assessment methods. We included controlled studies (ED cohorts vs. healthy controls) that applied at least one indirect bias assessment method. By providing a systematic overview of the relevant literature in the field, we aimed to examine the evidence based on which implicit biases or discrepancies between implicit and explicit biases may explain disordered eating patterns in patients with EDs. Findings may help to adequately design and apply future interventions based on implicit bias modification.

## Materials and methods

In conducting this updated systematic review, we followed the guidelines of the PRISMA statement (preferred reporting items for systematic reviews and meta-analyses) [30].

### Search strategy

To carry out the systematic part of this review, we conducted a systematic search (lastly performed on July 6th, 2020) by hand in the PubMed database (www.pubmed.gov) as well as in the Cochrane Central Trials Register using MeSH terms, keywords of interest, and their combinations. Exemplarily, the PubMed search included the following terms: ((((((((eating disorder) OR anorexia nervosa) OR bulimia nervosa) OR binge eating disorder) OR OSFED) OR EDNOS)) AND ((((((((((((indirect assessment) OR indirect test) OR indirect task) OR indirect method) OR implicit method) OR implicit task) OR implicit assessment) OR implicit association test) OR approach avoidance test) OR approach avoidance task) OR implicit association task) OR implicit test)) AND (((bias) OR implicit bias) OR implicit). We have additionally carried out a hand search for relevant articles cross-cited in search results and inspected reference lists to identify further studies of interest.

The studies were categorized into those examining implicit bias to food vs. body cues. Specifically, we addressed the following aspects:

1. Implicit biases in clinical (ED) cohorts: food cues
2. Implicit biases in clinical (ED) cohorts: body image cues

**Fig. 1:**
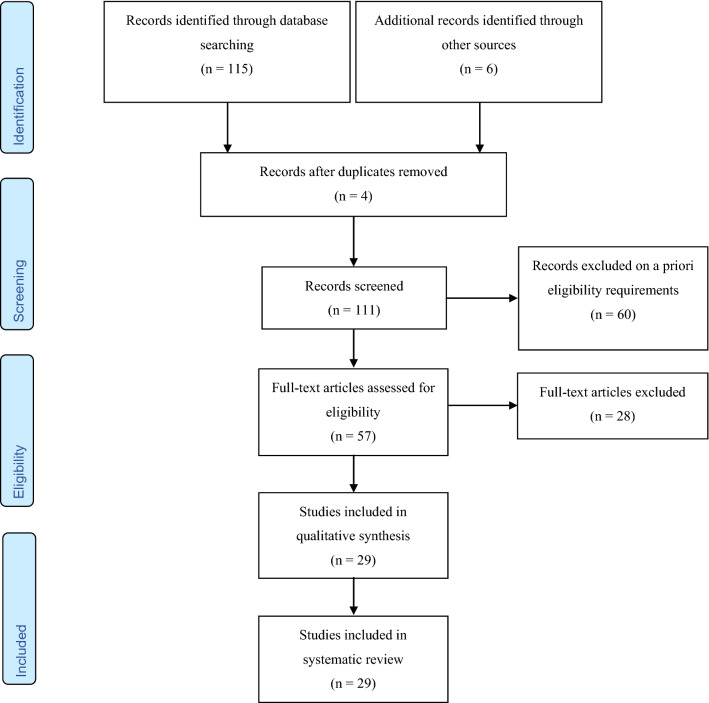
Search methodology according to the guidelines of the PRISMA statement (preferred reporting items for systematic reviews and meta-analyses) [30]

A consort diagram describing the search and selection stages is displayed in Fig. 1.

### Eligibility criteria

Eligibility criteria were based on the PICOS taxonomy according to the PRISMA statement [30], defining criteria for each of the five domains, i.e., participants (P), interventions (I), comparators (C), outcome (O) and study design (S). We included studies performed in a defined population P (patients with eating disorders) and assessing implicit bias to food and body cues as outcome O. We also only included analytic studies, thus, experimental (e.g., RCTs) and observational analytic studies (e.g., cohort studies) with an intervention I. We also only included studies that examined the rates of outcomes in a comparison group C. A two-step process was used to evaluate the results of the literature search. First, the titles and/or abstracts of all publications mentioned above were independently reviewed by G.P. and A.D.S.-H. prior to the retrieval of full-texts. Second, articles identified eligible for full review were further screened based on specific eligibility criteria. The final decision to include studies in the present work was based on the following criteria: (I) studies including a clinical cohort, thus patients diagnosed with an ED. Studies performed in nonclinical cohorts, e.g., undergraduate students, were not considered for inclusion. However, we discussed studies in nonclinical cohorts with relevant results in this review as well, (II) studies reporting implicit biases using at least one indirect experimental task, (III) studies including a healthy control group or another control condition, e.g., restrictive vs. binge/purge AN, (IV) studies including adults over 18 years of age, and (V) articles written in English. Differences of opinion between both authors were resolved through consensus.

### Participants

We included studies in adult patients diagnosed with an ED, and only studies including a control group.

### Interventions

A great variety of indirect tests to assess implicit biases have been developed and are currently in use. Of these, the implicit association task (IAT) is considered the prototype [31]. In this test, participants are presented with cues (mostly words) from 4 different categories (two category pairs, e.g., “sweets/vegetables” and “positive/negative”) and are asked to associate these cues as fast as possible using predefined response options (e.g., by pressing a left key in the case of a cue belonging to “sweets” or “positive” and a right key for “vegetables” or “negative” or vice versa in subsequent runs). As a result, responses to “compatible” associations are faster compared to “incompatible” ones and display biases. Similarly, in the approach avoidance task (AAT), participants are asked to either push or pull target cues using a joystick or computer mouse, but responding to an irrelevant cue feature (e.g., picture format) and not to the cue itself; the direction of motion gives participants the impression of actually approaching cues by pulling them towards them or avoiding cues by pushing them away [32]. Again, compatible cue x direction of motion pairs led to faster responses compared to incompatible pairs and constitute implicit biases. We did not limit our review to just specific methodologies like the IAT or the AAT and describe additional indirect tasks to assess implicit biases further below within the main part of this review.

### Comparators

We have decided to only include studies that have compared a (non-clinical) control group or control condition.

### Outcome

Implicit biases to food and body-related cues were the outcomes in all studies included in this review.

### Study design

We included not only studies that were cross-sectional but also studies reporting longitudinal data on implicit biases, e.g., the change in bias following an intervention or ED-specific treatment. Both retrospective and prospective studies were considered; however, no retrospective studies were retrieved. Additionally, we included only controlled studies to be able to draw more adequate conclusions as to the presence or absence of implicit biases in ED cohorts and the inferences thereof with actual eating behaviors.

## Results

### Study selection

We identified 115 publications through our systematic search and 6 publications through other sources (Fig. 1). After screening, 57 publications remained for the full-text analysis. We then excluded 28 of these publications as they did not fulfill one or more of the eligibility criteria. In the end, 29 studies were included in this present systematic review of the literature. Reasons for exclusion were: studies in adolescents only (*n* = 5), no indirect assessment method of interest (*n* = 5), study in a non-clinical cohort (*n* = 10), lack of a control group (*n* = 6), study protocol (*n* = 2), and review (*n* = 2) (Fig. 1).

### Studies investigating implicit biases in clinical (ED) cohorts: food cues

We included *n* = 16 studies that examined implicit biases in ED cohorts employing food cues and fulfilled the eligibility criteria.

In the so-called affective priming paradigm (APM), two words are presented in quick succession and participants read the presented first word (“prime”) and are asked to respond to the second word (“target”). Congruent trials (e.g., palatable prime/positive target) are associated with faster responses than incongruent ones (e.g., palatable prime/negative target). In the study by Roefs et al. [33] using the APM, healthy controls showed an implicit bias for high palatable food cues compared to patients with AN [33]. Also using the affect misattribution procedure (AMP), a computer-based measure of implicit affect in response to disorder-specific cues, patients with AN showed significantly higher negative implicit bias for high- but not low-calorie food [34]. In line with these results, patients with AN and BN showed a higher negative implicit bias to food cues in the AMP compared to a control group [35]. Negative implicit biases to food were predictive of ED symptoms and ED-related behaviors in an assessment 4 weeks later [35].

Our group used the approach avoidance task (AAT) to test the hypothesis that an implicit avoidance bias for high-calorie cues would be found in patients with AN compared to healthy controls, who instead would display an approach bias for high-calorie cues [36]. As the main result, we found that healthy controls showed an approach bias for food cues independent of calorie content, as reflected by faster reaction times in the “pull” compared to the “push” condition; this bias was absent in the group of patients with AN [36]. Seibt et al. [25] examined the effect of food deprivation on the immediate valence and implicit bias to food in a small group of patients with AN and BN and controls using the affective Simon task (AST) [25]. Study participants had to either approach food pictures or avoid them by moving an animated manikin towards a food picture or away from it. The authors found that, regardless of the presence of an ED, the implicit approach bias to food was facilitated by hunger [25]. Finally, using similar procedures, Nejmeier et al. [37] compared the AST with food as a task-irrelevant stimulus against a Stimulus Response Compatibility (SRC) task with food as the task-relevant stimulus that could not be ignored; the authors found a reduced approach bias when food stimuli were task irrelevant but an increased avoidance of food when food stimuli were the task-irrelevant feature [37].

Changes in facial electromyographic activity, skin conductance and heart rate mirroring implicit responses were assessed in patients with AN and controls in the study by Soussignan et al. [38]. The authors exposed all participants to palatable food cues following a subliminal exposure to facial emotional cues (e.g., fear or disgust). Subliminal fear cues increased facial electromyographic activity in response to food in AN compared to controls [38]. Unconscious fear may, thus, increase the negative bias to food in patients with AN.

Despite lower implicit bias for palatable food in an IAT in a clinical ED group in the study by Mattavelli et al. [39], three sessions with transcranial direct current stimulation (tDCS) on frontal and occipito-temporal cortices were shown to increase implicit biases specifically to food cues compared to control women. This effect was specific for food but not body cues [39].

Cowdrey et al. [40] examined the motivational (wanting) bias to high- and low-calorie food at both an implicit and an explicit level in females at different illness phases of AN and healthy controls. The authors applied a forced-choice methodology, during which patients had to select from a pair of food cues the one that they most wanted to eat at that moment using a keyboard response. By covertly measuring reaction times to the food cues, interpreted as a degree of preference, patients were unaware of their implicit bias to food assessed on the task. The authors showed that patients with acute as well as weight-restored AN demonstrated significantly lower implicit wanting for high-calorie foods and higher implicit wanting for low-calorie foods; the opposite was the case in controls. Patients with acute AN reported significantly lower explicit wanting for high-calorie foods than the other groups [40].

Werthmann et al. [41] compared adults with AN to adults without AN applying the visual probe task. In this task, two pictures are presented next to each other, following a target cue replacing one of the pictures. Participants are asked to identify the position of the target cue. According to the theory behind the task, participants will respond faster to the target cue if it appears in the place of the picture that had captured their attention the most. Thus, response latencies display an indirect measure of attention. The authors showed that the patient group avoided maintaining attention on food vs. non-food cues [41].

Nonetheless, there is also deviating evidence. In the word stem completion task, participants are given the first few letters of a word and are required to complete the word as quickly as possible. This way, schema activation rather that activation of explicit knowledge is elicited. Patients with AN showed a strong explicit memory bias for words related to AN (e.g., thighs, thin, kilo, chocolate) compared to controls; however, no similar bias in the implicit word stem completion task was found in the study by Hermans et al. [42]. Applying the startle eyeblink modulation paradigm to assess appetitive and aversive responses in patients with EDs, Friederich et al. [43] found that patients with AN had a startle response to food cues that did not differ from controls [43].

In contrast to findings in AN [41], binge eaters with obesity exhibited higher latencies to disengage attention away from food cues in the visual probe task compared to obese participants without binge eating episodes [44]. In a previous study of our group, we found a significant avoidance bias to low-calorie food in patients with BED, not different to the bias displayed in the control group. To explain the surprising lack of differences between patients with BED and controls, we argued that biases underlying binges may not be captured adequately under laboratory conditions, thus outside the context of an actual bingeing episode [45]. Leehr et al. [46] followed a different approach, with facial electromyography recordings suggestive of automatic, rapid (implicit) responses, and reported different results. The authors examined normal-weight controls vs. overweight participants vs. overweight patients with BED. They found that all groups showed a negative implicit bias to food cues (compared to non-food cues). However, the groups reported an explicit positive bias to food cues (compared to non-food cues). The strength of the explicit bias was overweight + BED > overweight participants > normal-weight participants [46].

The double-blind randomized controlled trial by Brockmeyer et al. [47] compared, real, vs., sham “ABM” (approach bias modification) training sessions. Over the course of 4 weeks, participants with BN and BED were trained (or not) to avoid food cues. Training was determined by a higher number of avoidance over approach movements to food, while in the sham training participants had an equal number of approach and avoidance movements to food and non-food cues. A total of 10 training sessions were not able to influence approach and attention bias to food or actual food intake, although the real ABM condition was associated with more significant reductions in ED symptoms than sham ABM [47].

All studies presented in 3.2 are shown in Table 1.

**Table 1:** The table displays current research findings with regard to indirect measures and implicit biases in the field of food-related cues in patients with eating disorders. For details please refer to the main text

Publication	Participants	Purpose	Instruments (indirect measures)	Control group or condition	Main outcome
Implicit bias in clinical cohorts: food					
[42]	*N* = 12 inpatients with AN and *N* = 12 nondieting controls	To test the activation of anorexia-related concepts compared to unrelated concepts in patients with AN and controls	Implicit word completion test	Healthy controls	The data did not support an implicit memory bias for AN-related words in patients with AN
[33]	*N* = 22 patients with AN vs. *N* = 27 unrestrained lean controls (experiment 1), *N* = 27 obese participants vs. *N* = 27 unrestrained lean controls (experiment 2)	To test the sensitivity to the palatability of foods in patients with AN and obese participants compared to unrestrained lean controls	Affective Priming Paradigm	Un-restrained lean controls	In contrast to controls, patients with AN did not show a bias for palatable (low- vs. high-fat) foods (experiment 1). Obese participants showed a bias for low-fat over high-fat foods; this result was interpreted as the result of health concerns in the obese cohort (experiment 2)
[43]	*N* = 30 females with an ED (*N* = 15 AN, *N* = 15 BN) and *N* = 30 female controls	To examine appetitive and aversive responses to food and body cues in patients with EDs	Acoustically elicited Startle Eyeblink Modulation (SEM)	Healthy controls	Females with BN showed an appetitive response (startle inhibition) to food cues relative to neutral cues, while patients with AN showed a generalized failure to activate the appetitive motivational system
[38]	*N* = 16 females with AN, *N* = 25 female controls	To examine the influence of subliminal emotional processes on bias to food in AN	Subliminal exposure to facial expressions (happy, disgust, fear, and neutral faces) combined with facial electromyo-graphic activity, skin conductance, heart rate and videotaped facial behavior	Healthy controls	Subliminal fear cues increased the negative bias to food cues in patients with AN
[25]	*N* = 7 patients with BN and *N* = 13 patients with AN (study 3)	To examine how food deprivation influences the immediate valence of food cues and motivational bias for food cues	Affective Simon Task (study 3)	Healthy controls, both satiated and food deprived (study 3)	Food deprivation led to a more positive immediate valence of food items in the IAT and EAST. Approach bias was facilitated for participants tested before lunch compared to those tested afterwards, even in the ED cohort
[40]	*N* = 20 females with current AN, *N* = 22 females with weight-restored AN, *N* = 22 females with fully recovered AN and *N* = 41 healthy females as controls	To examine implicit and explicit wanting and liking in a group of acute AN and weight-restored AN compared with fully recovered patients with AN and controls	An implicit wanting reaction time measure in a forced-choice procedure	Healthy control group, weight-restored and fully recovered AN patients	Acutely underweight females with AN explicitly wanted high-calorie foods less than the other groups did. Both current and weight-restored groups with AN demonstrated significantly less implicit wanting for high-calorie foods and more implicit wanting for low-calorie foods; the opposite pattern was seen in healthy females
[34]	*N* = 25 females with AN (*N* = 9 with acute AN, *N* = 14 recovered from AN), *N* = 29 healthy control females	To test responses to low- and high-calorie cues in patients with AN	AMP	Healthy control group and control group recovered from AN	Patients with AN showed significantly higher negative implicit bias for cues of high- but not low-calorie food, indicating that they do not display negative implicit bias for all foods
[36]	*N* = 41 patients with AN and *N* = 42 controls	To assess explicit and implicit biases towards low- and high-calorie foods in patients with AN compared to controls	Implicit AAT	Healthy control group	Healthy controls showed an approach bias for food independent of calorie content; this bias was absent in the group of patients with AN
[46]	*N* = 61 Obese patients (with and without BED) and healthy controls	To examine implicit bias for food cues vs. non-food cues in obese participants with vs. without BED and controls	Facial electro-myography	Healthy control group	Despite higher explicit negative bias towards food in the group of obese patients with BED compared to the other groups, all groups under investigation showed a negative implicit bias towards food (vs. non-food cues) as assessed using facial electromyographic recordings
[45]	*N* = 24 obese patients with BED (OB-BED), *N* = 32 obese participants without BED, *N* = 25 healthy controls	To assess explicit and implicit biases towards low- and high-calorie foods in obese participants with BED (OB-BED), obese participants without BED, and controls	Implicit AAT	Healthy control group and group of obese without BED	Both the OB-BED group and healthy controls showed an avoidance bias for pictures of low-calorie food
[44]	*N* = 19 obese patients with binge eating and *N* = 23 participants without binge eating	To detect attentional bias (slower attentional disengagement) from unhealthy food in obesity in different stages of the attentional process	Computerized Visual Probe Task	Obese participants without binge eating as a control group	Obese patients with binge eating behavior displayed a more pronounced attentional bias towards food than participants without binge eating behavior
[35]	Patients with AN and BN (*N* = 92) and controls (*N* = 85)	To test if patients with EDs differ from controls in implicit biases to food and body cues and if these biases predict ED symptoms and behaviors over a 4-week period	AMP	Healthy controls	Patients with AN and BN showed a higher negative implicit bias to food compared to controls. Negative implicit biases to food were predictive of ED symptoms and ED-related behaviors in an assessment 4 weeks later
[39]	*N* = 21 females with AN, *N* = 13 females with BN, *N* = 2 females with EDNOS; *N* = 36 healthy control females	To examine the effect of transcranial current stimulation (tCDS) on implicit biases towards food and body cues	IAT	Healthy controls	Three sessions of tCDS led to an increase of implicit bias for food in the ED group. This effect was specific for food cues
[41]	*N* = 39 adults with AN, *N* = 34 adolescents with AN, *N* = 53 adult controls and *N* = 31 adolescent controls	To examine if attention bias to food differs across age and illness duration	Visual Probe Task with concurrent eye-tracking and response latency assessment	Healthy controls	All participants had a direction bias (i.e., heightened attention capture) for food, specifically for high-calorie food. However, adults with AN subsequently avoided maintaining attention (i.e., had a decreased duration bias) to food versus non-food cues compared to controls. Adolescents with AN showed significantly increased attention maintenance on food stimuli
[47]	*N* = 56 patients with BN and BED	To examine if Approach Bias Modification (ABM) may reduce implicit bias to food (i.e., approach bias) as well as binge eating and related symptoms	ABM modification training vs. sham training	Sham condition	ABM tended to result in greater reductions in ED symptoms than sham ABM. Food intake, approach bias, and attention bias to food remained unaffected
[37]	Patients with AN (*N* = 63), and a comparison group of adolescents without eating pathology (*N* = 57)	To investigate differences in patients with AN with regard to implicit vs. explicit biases towards food in two tasks applying food as task-irrelevant vs. -relevant feature	Affective Simon Task and Stimulus Response Compatibility task	Healthy controls	Patients with AN showed a reduced implicit approach bias towards high-calorie food

### Studies investigating implicit biases in clinical (ED) cohorts: body cues

We included *n* = 18 eligible studies that examined implicit bias to body cues in ED cohorts, including *n* = 5 studies that examined food and body cues and were already presented in “Studies investigating implicit biases in clinical (ED) cohorts: food cues” [34, 35, 39, 42, 43].

No differences in startle responses to thin female bodies were found between females with AN, BN, and controls in the study by Friederich et al. [43], a finding confirmed by Brockmeyer et al. [48] who found no differences between patients with AN and healthy controls in the implicit evaluation of emaciated bodies in an AAT. Interestingly, after replacing the face on body stimuli by the participant’s own face, patients with AN displayed an implicit bias for that manipulated body over the one carrying the standard face [48].

Spring and Bulik [34] hypothesized that patients with AN would display significantly higher negative implicit bias to overweight and significantly higher positive implicit bias to underweight in an affect misattribution procedure (AMP). As expected, patients with AN showed significantly stronger negative implicit bias to overweight cues [34]. In line with these results, patients with AN and BN showed more negative implicit bias to average body stimuli compared to a control group [35].

Smith et al. [49] determined whether patients with AN associate emaciation with beauty using the lexical decision task, applied to examine biases to emaciated compared to underweight bodies. The authors hypothesized that women with AN primed with emaciation would recognize words associated with “beautiful” faster than women in the control group who were primed with emaciation as well as faster than women with AN primed with thinness. Women with AN showed a stronger association between emaciation and beauty than control women. Additionally, ED symptoms were found to significantly predict the robustness of the association between emaciation and beauty, but, paradoxically, also between emaciation and ugliness [49]. Other authors have shown that negative implicit bias to overweight—rather than positive bias to ultra-thin role models—appears to be a key issue in AN [50].

These results are in keeping with previous results obtained using affective priming in patients with AN, BN and healthy controls; Blechert et al. [51] examined explicit and implicit associations between shape/weight and the participants’ self-evaluation and found that both ED groups showed significantly more pronounced implicit bias than controls in terms of associating shape/weight concerns with self-evaluation domains [51].

Parling et al. [52] compared implicit pro-thin and anti-fat biases to the self and others in patients diagnosed with AN/subthreshold AN and healthy controls using the Implicit Relational Assessment Procedure (IRAP). The IRAP assesses hypothesized a priori established verbal relations (e.g., same or opposite) between sample (e.g., pleasant) and target cues (e.g., love). As in other tasks of this kind, faster responding is to be expected when same is required, indicating congruence between sample and target cue and, thus, a bias. The clinical cohort showed an implicit pro-fat bias to others and stronger anti-fat bias to the self compared to controls. These findings were related to the over-evaluation of weight and shape in the clinical group [52].

Patients with AN often have a distorted perception of their own body and show deficits in interoception and haptic perception; conflicts in visual and tactile integration might be the case. Case et al. [53] investigated mechanisms leading to body image distortion in AN using the size-weight illusion (SWI) that is based on the implicit premise that small objects are heavier than large objects (of the same weight). The authors found that patients with AN had a significantly lower SWI compared to controls, although they had no difficulties to discriminate weight. Because the SWI is impacted by visual appearance, this result was presented as evidence towards a disintegration of visual and proprioceptive input in AN, in terms that patients with AN rely less on visual input but more on proprioceptive information when it comes to judging weight [53].

Using an ineffectiveness induction procedure followed by an appearance-related word stem completion task as the indirect assessment of implicit bias, McFarlane et al. [54] tested the body displacement theory which claims that patients with EDs will project aversive feelings about themselves onto their body. Patients with EDs who were made to feel ineffective had indeed elevated implicit appearance/body concerns compared to unrestrained and restrained eaters in the control group who did not display similar effects [54].

In a small study, Watson et al. [55] examined reward for face and body cues of others and attention using eye-tracking techniques in weight-restored females with AN and controls [55]. While the two groups of participants were similar in ratings of attractiveness, the group with AN was less likely to look at women’s faces when the body was presented as well and less likely to look in the eye region when faces alone were presented.

Brauhardt et al. [56] performed a study with obese-only participants, obese patients with BED (OB/BED) and controls to investigate associations between implicit and explicit weight bias in these groups. Higher explicit weight bias was found in the OB/BED group compared to both the other groups. However, the OB/BED group and the control group showed an equally strong implicit weight bias in the IAT, while the obese-only participants did not [56].

Khan and Petroczi [57] applied computerized tests measuring subconscious normative Ideal Body Image (IBI), Personalized self-identification Body Image (PBI) associations and Food Preferences (FP). Patients with EDs showed significantly stronger bias to a thin body image and stronger self-identification with a thin body image compared to healthy women. No differences were found in preferences to food. This study demonstrated that implicit biases with regard to body image were more adequate to distinguish patients with ED than food-related tasks, therefore suggesting that body image is at the core of disordered eating psychopathology [57].

Biases to the own body were assessed using the Mental Motor Imagery Task (MMI) in the study by Purcell et al. [58]. Participants were asked to imagine making a movement along their body and then to actually perform the movement. The mental image of one’s own body (body schema) was evaluated comparing the time needed to perform the two actions. Purcell et al. demonstrated that participants with ED had a distorted body schema, implicitly assuming that sensitive to control body parts were larger than they actually were [58].

Finally, in a recent study using an IAT, Izquierdo et al. [59] found fat-phobic and non-fat-phobic patients with AN, as well as underweight restrictive eaters and healthy controls to all display implicit negative bias towards underweight models [59]. Korn et al. [60] also examined patients with AN who self-reported fear of gaining weight and patients who denied it, and found a disparity between explicit statements and results in an indirect bias assessment task (implicit conjoint analysis, CA) to be present in non-fat-phobic patients, thus providing evidence that an implicit drive for thinness might as well exist in patients with AN who explicitly deny fat phobia [60].

All studies presented in “Studies investigating implicit biases in clinical (ED) cohorts: body cues” are shown in Table 2.

**Table 2:** The table displays current research findings with regard to indirect measures and implicit biases in the field of body-related cues in patients with eating disorders. For details please refer to the main text

Publication	Participants	Purpose	Instruments (indirect measures)	Control group or condition	Main outcome
Implicit bias in clinical cohorts: body image					
[43]	*N* = 30 females with an ED (*N* = 15 AN, *N* = 15 BN) and *N* = 30 female controls	To examine appetitive and aversive responses to food and body cues in patients with EDs	Acoustically elicited Startle Eyeblink Modulation (SEM)	Healthy controls	There were no significant differences between groups regarding responses to body cues
[50]	*N* = 35 females with restrictive AN and *N* = 35 matched normal weight controls	To examine whether an ultra-thin ideal or negative bias for overweight might be the motivation behind pathological restriction in AN	modified Affective Priming Test	Healthy control group	Unlike the control group, patients with AN did not show a positive bias for the ultra-thin body shape. Patients showed a negative bias for overweight both on the implicit and explicit level
[55]	*N* = 11 females with weight-restored AN, *N* = 11 female controls	To investigate the implicit reward value of social stimuli for AN-patients	Eye-tracking	Healthy controls	Results showed that the faces of other females elicit approach behavior in control females but not in weight-restored AN. Females with Weight-restored AN hyperscanned emaciated bodies but explicitly reported that underweight bodies were less attractive than normal-weight body cues
[54]	*N* = 33 restrained eaters, *N* = 61 unrestrained eaters, *N* = 26 patients with ED	To test the body displacement theory by means of an ineffectiveness induction procedure and a body dissatisfaction measure	Appearance-related Word Stem Completion Task	Non-clinical cohort (unrestrained and restrained eaters) as a control group, control condition with self-esteem conducive memories	Patients with EDs who were made to feel ineffective reported more implicit appearance/body concern than participants in both control conditions
[51]	*N* = 20 patients with AN, *N* = 20 patients with BN, *N* = 28 controls	To examine associations between weight/shape concerns and self-evaluation		Affective Priming paradigm	Shape/weight concerns and self-evaluation were linked in the ED groups but not in controls
[52]	*N* = 17 females with AN, *N* = 17 female controls	To compare weight-related beliefs in women with AN and sub-threshold AN versus healthy women, while at the same time exploring the relation between bias for oneself and for others	Implicit pro-thin and anti-fat bias towards self and others using the Implicit Relational Assessment Procedure (IRAP)	Healthy control group	Patients showed an implicit pro-fat bias for others, but stronger anti-fat bias for the self
[53]	*N* = 10 females with AN and *N* = 10 healthy controls	To examine the mechanisms underlying body image distortion in AN	Size-Weight Illusion (SWI)	Healthy control group	Patients with AN exhibited a markedly reduced SWI relative to controls, even though their ability to discriminate weight was unaffected
[34]	*N* = 25 females with AN (*N* = 9 with acute AN, *N* = 14 recovered from AN), *N* = 29 healthy control females	To assess responses towards overweight and underweight In patients with AN	AMP	Healthy control group and control group recovered from AN	Patients with AN showed significantly more negative implicit bias for overweight cues
[49]	*N* = 30 females with AN, *N* = 29 healthy control females	To determine if patients with AN associate emaciation with beauty	Lexical Decision Task	Healthy control group	Females with AN had a stronger association between emaciation and beauty than control females. Further, ED symptoms were found to significantly predict the strength of the association between emaciation and beauty. Paradoxically, also the association between emaciation and ugliness was stronger in patients with AN than in controls
[56]	*N* = 26 obese patients with BED, *N* = 26 obese-only participants, *N* = 26 healthy norm weight controls	To examine explicit and implicit self-esteem in BED compared to an obese-only and a control group	self-esteem IAT	Healthy control group and group of obese without BED	Levels of implicit self-esteem were found to be lower in obese patients with BED, as well as in the obese-only group when compared to healthy control
[57]	*N* = 53 women with an ED (BN, AN, EDNOS), *N* = 41 females at risk for an ED, *N* = 23 healthy females	To assess bias to foods as well as self-esteem, levels of body dissatisfaction and body image perception in women with EDs compared to healthy controls	Computerized online tests assessing bias with regard to subconscious normative Ideal Body Image (IBI), Personalized self-identification Body Image (PBI) and Food Preferences (FP) using IATs and AAT	Healthy control group	Patients with ED showed significantly stronger biases for thin body images and stronger self-identification with thin body images compared to healthy females. No differences were found in food bias
[35]	Patients with AN and BN (*N* = 92) and controls (*N* = 85)	To test if patients with EDs differ from controls in implicit biases to food and body cues and if these biases predict ED symptoms and behaviors over a 4-week period	AMP	Healthy controls	Patients with AN and BN showed a higher negative implicit bias to average body cues compared to controls
[58]	*N* = 42 inpatients with ED, *N* = 40 healthy controls	To interrogate the body schema in patients with ED by assessing participants’ mental image of their body (i.e., body schema)	Implicit Mental Motor Imagery (MMI) task	Healthy controls	Participants with eating disorders consider themselves to be larger than they truly are
[39]	*N* = 36 women with ED, *N* = 36 healthy controls	To assess the effect of transcranial direct current stimulation (tDCS) on implicit food- and body-related biases	IAT (body-pictures)	Healthy controls and sham-tDCS	tDCS on frontal and occipito‐temporal cortices showed no effects with regard to body stimuli
[60]	*N* = 30 fat-phobic and *N* = 7 non-fat-phobic women with AN, and *N* = 29 healthy control women	To examine if explicit and implicit evaluations of weight gain are congruent	Conjoint Analysis (CA)	Healthy controls	Correlation between explicitly assessed drive for thinness and implicit CA score was low
[48]	Study 1 (foreign face on body avatar): *N* = 40 women with AN and *N* = 40 healthy women; study 2 (participants’ own face on body avatar): *N* = 39 women with AN and N = 38 healthy women	To investigate implicit evaluations of thin and normal-weight bodies with and without identification with the respective body-avatar	AAT using a body avatar with a standard face or the participants’ face	Healthy controls	No differences in implicit approach bias towards thin and normal-weight bodies (study 1). Patients with AN displayed an implicit bias for the manipulated body over the one carrying the standard face
[59]	*N* = 39 fat-phobic (FP) and *N* = 13 non-fat-phobic (NFP) women with AN, *N* = 10 women with avoidant/restrictive food intake disorder, *N* = 32 healthy controls	To assess possible differences in explicit and implicit biases between fat-phobia and non-fat-phobia as core symptoms of eating pathology	IAT using pro-dieting statements and body image-based IAT	Healthy controls	FP- and NFP-AN displayed a positive bias to pro-dieting statements. In the body image-based IAT all groups showed a negative bias to underweight models, although healthy controls had a significantly stronger negative bias than patients with FP-AN and NFP-AN

## Discussion

We carried out a systematic search of the literature and identified studies presenting implicit (automatic, rapid, non-verbal) biases to food and body cues in the context of EDs. Twenty-nine studies met the eligibility criteria and were included in this review. We identified 16 eligible studies using food cues and 18 eligible studies with body cues. Despite the methodological heterogeneity and considering conflicting results, some main observations may be enunciated in an attempt to synthesize current findings and conclusions.

Differences between patients with EDs and healthy controls regarding implicit biases to food are found across studies. Repeated findings showed a lack of incentive salience to food (in terms of reduced bias to high-calorie food or food in general) in patients diagnosed with AN [36]. However, restrained eaters among healthy controls were found to display a bias to high-calorie food [14]. Such findings may have several implications. Despite the methodological heterogeneity between studies, they might explain the ability of patients with AN to explicitly control their food intake, although it is not clear, whether they describe predisposing vulnerability factors or disorder sequelae. Then, these findings may point towards different underlying mechanisms between patients with AN on the one side and attempts to restrict food intake in otherwise healthy controls on the other, although this assumption cannot provide inferences on aspects of cause/effect and needs to be further confirmed. Interestingly, healthy participants classified as high food cravers showed stronger approach bias to food [61], a finding that resembles findings in patients with BED [44]. The only study assessing the implicit bias to food in different stages of AN [40] showed that implicit bias to food in AN appears to be independent of weight status. In contrast to AN, implicit biases to food are understudied in BN. Disinhibited food intake in terms of binge eating might, however, be associated with implicit attentional biases to food in binge eaters [44]. It has also been suggested that implicit bias underlying binge eating episodes may not be captured adequately under laboratory conditions, thus outside the context of an actual bingeing episode [45].

Studies on implicit biases to body cues in patients with EDs also consistently show differences to controls. The available results suggest that the general pro-thin/anti-fat implicit bias previously found in IAT studies [19, 62] might be the result of pro-thin implicit bias to the self. As shown further above, there is also evidence that the underlying implicit bias that may negatively impact eating behaviors might be related to body image rather than to food per se [57], although this aspect requires replication. Surprisingly, studies in non-clinical populations have consistently shown implicit anti-fat and pro-thin bias in investigations with body cues [19]. Thus, in contrast to studies applying food cues, the approach bias to body cues seems to run in parallel in both healthy controls and cohorts with EDs. Furthermore, implicit bias to the thin ideal seems to be associated with the occurrence of ED-related symptomatology, and differences between non-clinical and clinical cohorts seem to lie in the grade of severity. Accordingly, ED-related symptoms were found to predict the strength of the implicit association between emaciation and beauty.

The variety in methodologies does not allow direct comparisons between studies, e.g., while in the study by Spring and Bulik [34], using the affect misattribution procedure, a negative bias only to high-calorie food was observed, indicating that patients with AN do not display negative implicit bias for all foods, other authors, using the approach avoidance task (e.g., [36]), found a negative implicit bias to food independent of calorie content. Thus, while differences in methodology do not permit generalization of results, the experimental paradigm per se might be considered to have an impact on the assessed bias. Similarly, the choice of body cues (emaciated, thin, normal weight, or overweight bodies) seems decisive for the identification of bias, and patients with AN hyperscan emaciated bodies while paying less attention to the face, as presented further above [55].

Implicit and explicit biases are capable of influencing behavior independently of each other [63]. Regarding food choice, a consistent and predictable relation between implicit and explicit biases and behavior remains inconclusive [64]. Some studies found relations between implicit—but not explicit—biases and spontaneous snack selections, while others found no such relation [64]. Implicit biases predict food choice when individuals have a low cognitive capacity (e.g., being distracted or emotional after watching an upsetting film) or when there is low inhibitory control (e.g., high levels of impulsivity) [11, 65, 66]. Low inhibitory control heightens the impact of implicit food biases on overeating [67–69] and is associated with the ingestion of higher amounts of high-calorie food [70, 71] the failure of diets [72], and even with obesity [70, 73–76]. Other authors found that implicit affective bias was a significant predictor of snack choice at low, but not high levels of ED symptomatology [76]. Given the prior inconsistencies in the strength of the relation between implicit bias and spontaneous eating behavior [64], the study by [76] suggests that ED symptomatology may moderate the strength of the relation. Possibly, elevated self-control, as in some types of EDs, reduces the impact of implicit affective biases on behavior [77]. Overall, there is an ambiguity remaining. The study by Goldstein et al. [78] showed that neither implicit nor explicit biases alone predicted disinhibited eating and that there was no mediating effect of impulsivity on implicit biases to predict eating behavior [78]. This finding confirmed a previous study showing that implicit biases were poor predictors of actual behavior [79].

### Bias trainings

On the behavioral level, implicit biases were a predictor of eating behavior at low levels of ED-related symptomatology. While treatment effects on implicit biases cannot be distinguished due to the lack of respective studies, there is limited evidence that biases are modifiable. This has led to the implementation of implicit bias trainings. Such trainings are up to now no established options for modifying eating habits or for the management of EDs. Prior to designing and providing implicit bias trainings, it is important to further examine the direction (approach vs. avoidance) as well as the specificity of bias (e.g., by testing disorder-related against neutral cues and involving healthy controls).

To facilitate behavioral modifications, research groups applied repeated response inhibition and were able to demonstrate reductions in the valence as well as the positive bias to cues [80–86]. Such interventions were mostly tested within the context of addictive behaviors and were proven effective, e.g., in modifying implicit approach biases to alcohol in patients with alcohol dependence [86]. These types of interventions to modify implicit biases may thus further diversify standard psychotherapeutic approaches.

It has been feasible to prompt individuals to eat less and/or healthier by training them to respond to temptations with an automatic inhibition movement [87–90]. Training of food-related inhibitory control appears to be especially effective for individuals with a strong urge to consume specific foods [81, 82, 87, 89]. In this sense, food-associated inhibitory control was practiced in a group of chocolate lovers who then consumed significantly less chocolate in an alleged taste test compared to participants who had not undergone the chocolate/no-go training [87]. Similarly, both implicit bias as well as craving for chocolate decreased following a training in which participants had to associate words of avoidance with chocolate pictures [91].

Preliminary evidence suggests that trainings using the AAT can modify implicit biases to food in healthy participants [92, 93]. Ferentzi et al. [94] trained obese participants to make avoidance movements in response to high-calorie, unhealthy food cues and approach movements in response to cues representing a healthy lifestyle, while a control group received sham training. The approach avoidance bias improved in the active training group compared to the control group, an effect that even generalized to novel, untrained food cues. No training impact was found on ED-related questionnaires or the BMI [94]. On the other hand, there are also studies failing to find effects. In the study by Becker et al. an AAT training in normal-weight individuals did not change the implicit bias to food, at least not following a single session [95]. Using a brief evaluative conditioning intervention, food intake was found to be malleable, but this effect was only observed among individuals with low inhibitory control [96]. Combined trainings of implicit bias modification and inhibition control seem to have an additional impact on implicit bias to unhealthy food: participants who were both trained to indirectly avoid pictures of unhealthy food and to inhibit responses to them in a go/no-go task showed less implicit bias (liking) to food in an IAT, while each training condition alone did not alter implicit biases in this study. Training did not affect explicit food choice or intake [97].

There are also training studies of implicit bias to food in (sub)clinical cohorts, although only one recent study by [47] fulfilled the eligibility criteria of inclusion in this review (e.g., presence of a control condition) [47]. In a previous study by the same authors in participants with subclinical BN and high levels of self-reported food craving, indirect avoidance movements to visual food cues were practiced in ten 15-min sessions over a 5-week course. The authors examined the effect of training on craving, ED symptoms, and bias to food as well as a possible generalization to other biases (i.e., attentional bias to food cues). At baseline, participants showed approach and attentional bias to high-calorie food that were significantly reduced and turned into avoidance biases after the training. Participants also reported pronounced reductions in food craving and ED symptoms [98]. This study added to the evidence suggesting that effects of a training targeting one specific (cognitive) bias may generalize to another [99]. Boutelle et al. [100] trained females with binge eating behavior to focus their attention away from food cues by reinforcing attention to the neutral word of a presented word pair which included one food-related and one neutral word. Repeated training sessions at least twice a week over 12 weeks led to reduced binge episodes, decreased weight, BMI and ED symptoms [100]. These results provide evidence for distinct clinical effects of attentional bias modification trainings. Due to the lack of a control group, it remains unclear if this training improved executive control of attention in general. The long-term efficacy of such trainings is still unknown.

Apart from explicit mechanisms of cognitive modification and emotion regulation, novel implicit trainings conceived as add-ons to classical psychotherapy are important to develop. Reducing discrepancy around unhealthy foods could be accomplished by either minimizing positive implicit bias (e.g., through associative priming or evaluative conditioning) [64, 101] and/or by minimizing negative explicit bias (e.g., decreasing rigidity and designations of so-called “forbidden foods”). Interventions aiming at reducing impulsivity (e.g., through computerized inhibitory control trainings) may also reduce the likelihood of eating in the presence of unhealthy foods [81]. In the case of AN, for example, implicit mechanisms of self-regulation would aim at attenuating the inhibitory, executive prefrontal control in patients with AN including automatic, conditioned reaction patterns by modifying dysfunctional incentive structures (i.e., increasing the salience of food-related cues). The anticipated outcome would then be a cutback on avoidance behaviors and the buildup of a stance over approach behaviors.

## Limitations

Several limitations should be considered when interpreting the results of the studies presented in this work. Relatively small samples sizes limit the generalizability of findings. Additional research is needed to clarify the replicability of implicit biases, due to the fact that most findings are singular, cross-sectional findings by different research groups. As most studies were cross-sectional, no conclusions about cause/effect can be drawn. Further longitudinal studies are necessary to examine the unique predictive validity of implicit vs. explicit bias on eating behaviors and in eating disorders. Finally, controlled bias modification (training) studies are still scarce. Only repeated and independent evaluations may avoid premature closure about the magnitudes of effects and potentially effective interventions. On the other hand, an overemphasis on repeating experiments could provide an unfounded sense of certainty when findings rely on a single approach. Multi-method approaches, including several indirect assessments of implicit bias are therefore needed. Finally, studies examining implicit biases to cues other than food or body cues (affect, learning, etc.) were excluded from this review, mostly due to the fact that single findings but no systematic investigations are published so far.

## Future directions

There is a need for novel, neurobiologically founded strategies intervening on the implicit (pre-verbal) level in addition to classical psychotherapy. A psychotherapeutic treatment that only accounts for behavioral change by means of the conscious influence on the self (e.g., cognitive strategies) does not take into account that self-regulation may as well be generated through the implicit and automatic route of processing. This may explain why individuals are often not capable to steer their behavior according to their own set goals [8, 68]. As proposed by the dual-process theories, the slower and complex reflective processes are not able to take effect early enough, to impact upon automatically generated processes [102].

Specific interventions relating to relevant aspects of implicit self-regulation may diversify our current psychotherapeutic approaches to EDs. The aim would be to transform explicit regulation into a more implicit and resource-based process, going along with decreased drain of cognitive resources and thus increased odds of successful implementation of new behaviors [103, 104]. Neurofeedback arises as a procedure that may facilitate the explicit regulation of otherwise involuntary (implicit) neural function [101, 105]. Frequent utilization of explicit strategies may, with time, automatize the initiation of more implicit processes. Implicit regulation mechanisms may be selectively targeted, e.g., in the form of training of automatic appraisals; a training of automatic appraisal was capable of influencing the interpretation bias in novel situations and strengthen the self-confidence of participants [106]. Technological innovations are likely to be instrumental in future empirical work to develop and evaluate effective trainings for appetitive behaviors [107]. Implicit bias trainings need to be further developed, standardized, delivered by skilled staff and continuously re-designed considering ongoing evidence-based practice. Future research may identify determinants of efficacy that may allow to choose an implicit bias intervention based on patients’ individual characteristics. Long-term efficacy is another serious challenge within this context.

- What is already known on this topic: Explicit and implicit biases towards food- and body-related cues have been found to differ between patients with eating disorders and healthy individuals. There have been attempts to therapeutically influence explicit and implicit biases.
- What this study adds: A systematic review on implicit response biases to food- and body-related cues in eating disorders.
